# Relevance of the Axis Spermidine/eIF5A for Plant Growth and Development

**DOI:** 10.3389/fpls.2016.00245

**Published:** 2016-03-02

**Authors:** Borja Belda-Palazón, Carla Almendáriz, Esmeralda Martí, Juan Carbonell, Alejandro Ferrando

**Affiliations:** Instituto de Biología Molecular y Celular de Plantas, Consejo Superior de Investigaciones Científicas-Universidad Politécnica de ValenciaValencia, Spain

**Keywords:** spermidine, eIF5A, hypusine, translation, polyproline

## Abstract

One key role of the essential polyamine spermidine in eukaryotes is to provide the 4-aminobutyl moiety group destined to the post-translational modification of a lysine in the highly conserved translation factor eIF5A. This modification is catalyzed by two sequential enzymatic steps leading to the activation of eIF5A by the conversion of one conserved lysine to the unusual amino acid hypusine. The active translation factor facilitates the sequence-specific translation of polyproline sequences that otherwise cause ribosome stalling. In spite of the well-characterized involvement of active eIF5A in the translation of proline repeat-rich proteins, its biological role has been recently elucidated only in mammals, and it is poorly described at the functional level in plants. Here we describe the alterations in plant growth and development caused by RNAi-mediated conditional genetic inactivation of the hypusination pathway in *Arabidopsis thaliana* by knocking-down the enzyme deoxyhypusine synthase. We have uncovered that spermidine-mediated activation of eIF5A by hypusination is involved in several aspects of plant biology such as the control of flowering time, the aerial and root architecture, and root hair growth. In addition this pathway is required for adaptation to challenging growth conditions such as high salt and high glucose medium and to elevated concentrations of the plant hormone ABA. We have also performed a bioinformatic analysis of polyproline-rich containing proteins as putative eIF5A targets to uncover their organization in clusters of protein networks to find molecular culprits for the disclosed phenotypes. This study represents a first attempt to provide a holistic view of the biological relevance of the spermidine-dependent hypusination pathway for plant growth and development.

## Introduction

Spermidine has been proven to be essential in yeast and mammals as a donor of the 4-aminobutyl group required for the enzymatic modification of a conserved lysine in the translation elongation factor eIF5A (Park et al., 1997). This function has been postulated as the most relevant for spermidine in eukaryotes, since active eIF5A is essential for cell proliferation and viability (Pagnussat et al., 2005; Chattopadhyay et al., 2008; Nishimura et al., 2012; Pällmann et al., 2015) and it may be also the underlying reason why spermidine synthase genes are required for the survival of *Arabidopsis* (Imai et al., 2004). The spermidine-mediated post-translational modification of eIF5A yields the unique residue hypusine in a two-step catalyzed reaction named hypusination (Park et al., 1993) that renders a fully active translation elongation factor (Saini et al., 2009; Park et al., 2010). Two enzymes act sequentially on eIF5A to carry out the hypusination process, namely the deoxyhypusine synthase (DHS) that forms the intermediate residue named deoxyhypusine, and the deoxyhypusine hydroxylase (DOHH) that finally delivers the mature eIF5A protein (Park, 2006). Recent studies have unmasked the crucial function of eIF5A within the ribosome as a sequence-specific translation factor required for translation of polyproline-rich proteins that may cause ribosome stalling (Gutierrez et al., 2013). The specificity of this function during translation seems to be highly conserved throughout evolution, since a bacterial structural homolog protein, EFP, also promotes translation of polyproline motifs (Doerfel et al., 2013; Ude et al., 2013). This represents a remarkable example of evolutive convergence for ribosomal function, since EFP becomes post-translationally activated on a lysine by totally different mechanisms but with a very similar chemical structure to hypusine (Navarre et al., 2010; Lassak et al., 2015). The sequence-specific activity of eIF5A during translation of polyprolines is helping to elucidate unallocated functions to spermidine as, for example, its requirement during yeast mating to facilitate the translation of polyproline-rich protein formin needed for the remodeling of the actin cytoskeleton (Li et al., 2014). A seminal bioinformatic study analyzed the evolutionary distribution of genes encoding polyproline-containing proteins whose translation is expected to depend on eIF5A/EFP (Mandal et al., 2014). The authors concluded that polyproline motifs are low in prokaryotic proteomes and their frequencies increase with the complexity of the eukaryotic organism, highlighting the importance of the association of polyproline-rich proteins with the axis spermidine/eIF5A during eukaryotic evolution. However, comprehensive studies by means of ribosome profiling performed in bacteria with EFP mutants have revealed that not every mRNA encoding polyproline-rich protein sequence *per-se* can be considered as a *bona fide* EFP client, but molecular features such as the location and the strength of the pause and the mRNA translational efficiency may all contribute to the final protein output (Elgamal et al., 2014; Woolstenhulme et al., 2015). A corollary derived from these studies is that expected functions for the axis spermidine/eIF5A based on computational data of polyproline encoded genes as potential eIF5A clients, must be correlated with functional arguments based on genetic approaches, as it has been done recently with mouse models (Pällmann et al., 2015).

To date the information of the eIF5A hypusination pathway in plants is scarce and limited to functional data based on overexpression or antisense approaches mostly performed in *Arabidopsis* (Duguay et al., 2007; Feng et al., 2007; Liu et al., 2008; Ma et al., 2010; Ren et al., 2013), for which a biochemical characterization of the pathway has been recently described (Belda-Palazón et al., 2014). However, the essential function of the pathway during plant embryo development, as it happens in mammals, demands a careful genetic dissection by means of conditional inactivation. Here we show the phenotypic consequences of conditional gene inactivation of the *Arabidopsis* rate-limiting enzyme DHS, encoded by a single gene, with the expectation that all three eIF5A protein isoforms will be equally inactivated upon *DHS* knockdown. We have uncovered novel functions for eIF5A during plant development and for adaptation to hormonal and nutritional cues, and we have also pursued a cautionary correlation with network-based bioinformatic analysis of proline repeat-rich proteins.

## Materials and methods

### Plant growth conditions and transformation

*Arabidopsis* wild type (Col-0) and transgenic plants were grown *in vitro* with solid MS medium containing 2.45 g/L MS salts (Duchefa, The Netherlands) and 6 mM MES buffer adjusted to pH 5.7 with KOH and solidified with 1% Phyto Agar. *Arabidopsis* seeds were sterilized and sown as previously indicated (Belda-Palazón et al., 2014) and stratified for 3 days at 4°C before being cultivated in growth chamber under long day (16 h light intensity 110 μmol m^−2^ s^−1^ and 8 h dark) or short day (8 h light intensity 110 μmol m^−2^ s^−1^ and 16 h dark) photoperiod conditions. 1% sucrose was added to the medium only when indicated. When counting the days of growth, day 0 always refers to the moment of transferring the plants to the growth chamber after stratification. Root growth assays were performed growing the plants *in vitro* on vertical plates, and using ImageJ software (Schneider et al., 2012) to quantify the main root length from the digital pictures taken at the indicated time. Root hair pictures were taken from 9 to 12-day-old plants grown *in vitro* with a digital camera connected to a binocular Nikon SMZ800. Transformations of *Arabidopsis* wild type (Col-0) and *ft-10* mutant (Yoo et al., 2005) with *Agrobacterium* strain GV3101/pMP90 containing constructs for *DHS* silencing (*siDHS*) were carried out according to the floral dip method (Clough and Bent, 1998). Selection of transgenic plants was achieved by adding 15 μg/mL hygromycin (Sigma, USA) to the medium. For each transformed genotype 10 independent transgenic T1 plants resistant to hygromycin were grown in the greenhouse to obtain T2 seeds. At least 60 T2 plants from each line were grown *in vitro* in the presence of hygromycin to select for transgenic lines with 3:1 ratio (resistant:sensitive). At least 15 T2 hygromycin resistant plants from each line with 3:1 ratio were grown in the greenhouse to obtain homozygous T3 seeds that were tested for 100% resistance to hygromycin *in vitro*. Six independent homozygous T3 transgenic plants were isolated and tested for dexamethasone-dependent silencing by reverse transcription and quantitative PCR (RT-qPCR) to finally select three homozygous independent transgenic lines. The homozygous *siDHS* transgenic lines were named *H, K* and *M* for the transformed wild type genotype and lines *3, 5*, and *18* for the transformed *ft-10* genotype.

### Plant treatments

For the dexamethasone (Sigma, USA) treatments on plant seedlings grown *in vitro*, a working concentration of 10 μM was used from a 100 mM stock solution in DMSO and the equivalent volume of DMSO was used as mock. The dexamethasone treatments *in vitro* were initiated either directly at the sowing step or as induction by transferring 7-day-old seedlings to fresh medium with dexamethasone for at least 3 days. Abscisic acid (ABA) plant hormone (Sigma, USA) was added to the medium at the indicated concentrations from a 10 mM stock solution dissolved in 50 mM Tris-HCl pH 8.8 and the mock solution contained the same solution without the phytohormone. The ABA experiments on root growth inhibition were performed with seedlings grown with or without 10 μM dexamethasone for 4 days and then transferred to fresh medium with or without 10 μM dexamethasone containing 10 μM ABA for additional 10 days when pictures were taken. The experiments of ABA effects on plant seedling establishment and germination were performed by sowing the seeds with or without 10 μM dexamethasone containing either 0.3 or 2 μM ABA, respectively. The treatments with D-Glucose (Fisher Chemical, USA) were carried out on seedlings, grown for 4 days in glucose-free medium with or without 10 μM dexamethasone and then transferred to fresh medium with or without 10 μM dexamethasone and 200 mM glucose for 10 additional days when pictures were taken. The NaCl (Fisher Chemical, USA) treatments were carried out by adding to the sowing medium a final concentration of 50 mM NaCl with or without 10 μM dexamethasone and pictures of plant seedlings were taken at the age of 14-day-old.

### Cloning procedures

To generate the constructs for conditional silencing of *Arabidopsis DHS* (*siDHS*), specific sequences located at the 3′ end of the gene were first identified with the use of the CATMA platform (http://www.catma.org). Primers including the *siDHS* sequences together with *attB* gateway recombination sites highlighted in italics were the following: *siDHS-Fwd*: 5′-GG*ACAAGTTTGTACAAAAAAGCAGGCT*GGCAAAGGATTA TTGACTACAAGAC-3′ and *siDHS-Rev*: 5′-GG*ACCACTTTGTA CAAGAAAGCTGGGT*TATACTGTGATGCTACCATAGCC-3′. The primers were used for PCR amplification of genomic DNA as template with *Pfx50*™ *DNA Polymerase* (Life Technologies, USA). The amplified PCR products were used to obtain the entry clone in pDONR-Zeo (Invitrogen, USA) by means of BP gateway recombination technology (Life Technologies, USA). The entry clone was subcloned by LR gateway reaction into the destination vector *pOpOff2* (Wielopolska et al., 2005) to yield the final binary construct that was introduced into *Agrobacterium* strain GV3101/pMP90 for plant transformation.

### Plant protein extraction, immunoblotting and gene expression by RTqPCR

All the protocols for protein extraction, 2D and 1D protein electrophoresis, immunoblot detection, and quantitative gene expression studies based on reverse transcriptase and quantitative PCR (RTqPCR) including housekeeping genes for normalization were described previously (Belda-Palazón et al., 2014). The primers used for qPCR not reported previously are indicated in Table 1 and were designed using the web tool pcrEfficiency (Mallona et al., 2011). Relative gene expression values and statistical analysis was performed using the REST 2009 software program (Pfaffl et al., 2002) available from Qiagen (http://www.qiagen.com). The anti-hypusine antibody ABS1064 from Merck Millipore (USA) was used according to the manufacturer.

**Table 1:** **Primers for qPCR**.

**Primer**	**Sequence**	**Reference**
*FT-Fwd*	5′-GGTGGATCCAGATGTTCCAA-3′	This study
*FT-Rev*	5′-ATTGCCAAAGGTTGTTCCAG-3′	This study
*FLC-Fwd*	5′-TGTGGATAGCAAGCTTGTGG-3′	This study
*FLC-Rev*	5′-TGGCTCTAGTCACGGAGAGG-3′	This study
*CO-Fwd*	5′-AACGACATAGGTAGTGGAGAGAACAAC-3′	Segarra et al., 2010
*CO-Rev*	5′-GCAGAATCTGCATGGCAATACA-3′	Segarra et al., 2010

### Tissue staining and microscopic analysis

Propidium iodide staining was performed by immersion of plant seedlings in a 10 μg/mL propidium iodide (Sigma, USA) solution for 10–15 min. After sectioning the root samples were mounted on a microscope slide and covered with a drop of distilled water. Confocal imaging was carried out with a Leica True Confocal Scanning (TCS) laser microscope. Visualization of red fluorescence was achieved by sample excitation with Helium-neon laser at 543 nm with a double band dicroic mirror (488/543) and spectral detection between 600 and 660 nm. Optical images of plant seedlings and roots were taken either with Nikon Eclipse E600 microscope equipped with digital camera DS-Ri1 or with binocular Nikon SMZ800 with incorporated digital camera Color View 12.

### Bioinformatic analysis

The analysis of protein interaction network for proteins containing the motif PPP or PPG, was performed with a similar approach to the work reported in mouse (Pällmann et al., 2015). We used a list of *Arabidopsis* genes encoding proteins with at least 2 PPP or 2 PPG motifs as described previously (Mandal et al., 2014). STRING database (http://string-db.org/) was used as a platform to identify protein-protein interaction networks (Szklarczyk et al., 2015) that were later visualized using the software Cytoscape (Shannon et al., 2003) version 3.2.1 (http://www.cytoscape.org/) with the plugins MCODE for cluster analysis (Bader and Hogue, 2003) and BiNGO for gene ontology description (Maere et al., 2005). Network topology parameters were obtained with the Network Analyzer (Assenov et al., 2008) built in the Cytoscape software tool and with the CytoNCA plugin (Tang et al., 2015).

## Results

### Generation and validation of transgenic *Arabidopsis* plants designed for the conditional silencing of the gene *DHS* encoding the enzyme deoxyhypusine synthase

**Figure 1:**
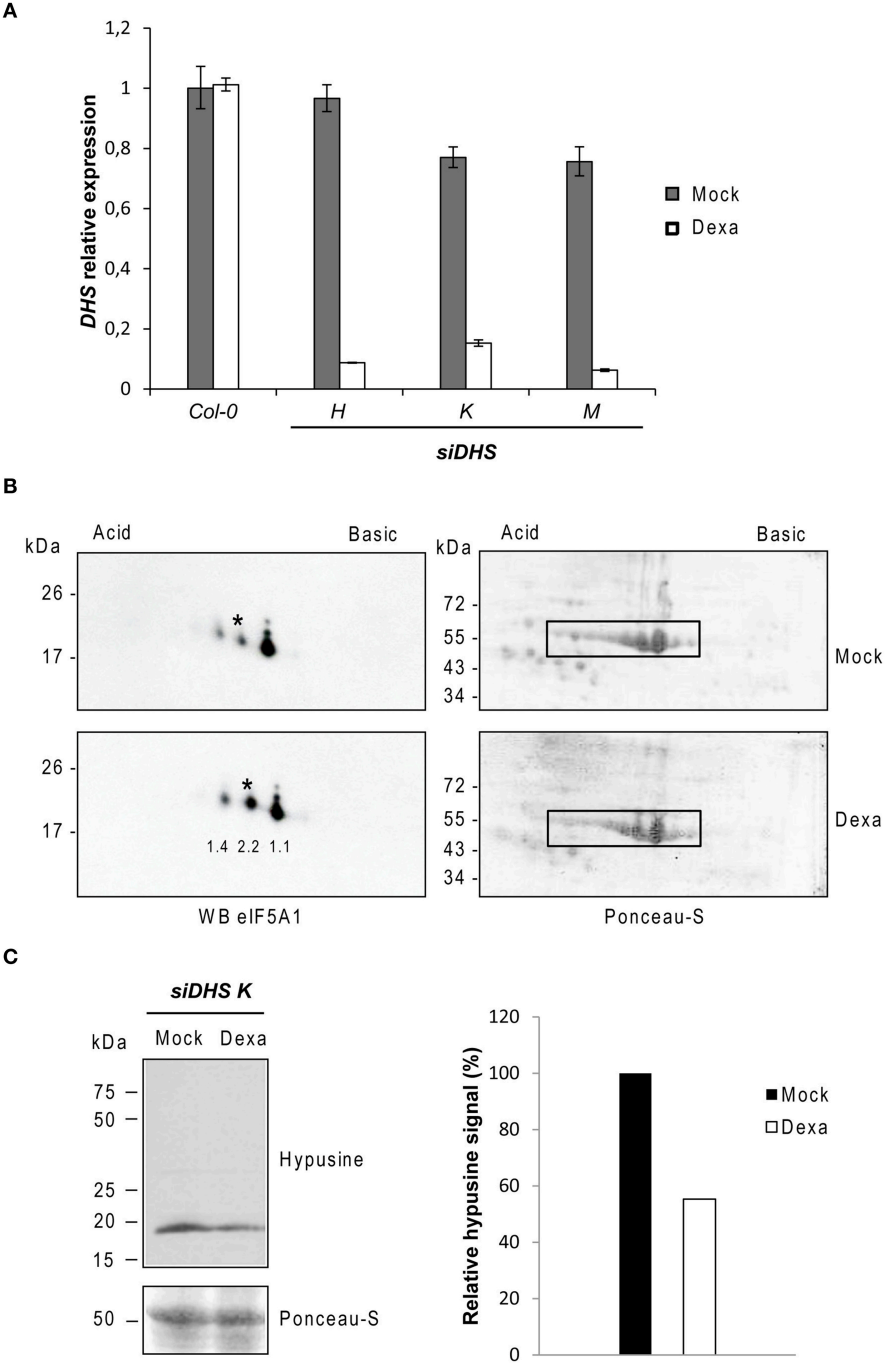
**Molecular characterization of *siDHS* transgenic plants for conditional *DHS* silencing**. **(A)** *DHS* gene expression relative to the mock Col-0 sample was carried out by RTqPCR with 10-day-old plant seedlings treated with (dexa) or without (mock) dexamethasone. Error bars represent standard deviation from three independent technical replicates. **(B)** 2D-electrophoresis/western blot analysis with anti-eIF5A1 antibody (left panels) was performed with the same amount of 35 μg total protein as shown by Ponceau staining (right panels) from plant seedlings treated or not with dexamethasone. The western blots represent a typical result of three independent biological replicates. Asterisks indicate the non-hypusinated eIF5A1 proteoform. The numbers below each spot on the immunoblot of the Dexa panel indicate the fold-change increase for every spot quantified by ImageJ analysis relative to the Mock panel. Both Dexa and Mock values were previously normalized to the Rubisco signals highlighted with a rectangle in their respective Ponceau staining panel. **(C)** On the left, SDS-PAGE/western blot analysis with anti-hypusine antibody is shown in the upper panel and Ponceau staining of the same blot in the lower panel. The bar diagram on the right shows the quantification of hypusine signal that was determined by measuring with ImageJ the band intensity relative to the Ponceau staining of the Rubisco protein in the same lane and plotted relative to the mock sample. The result was confirmed with two different biological replicates.

We selected the *DHS* single gene encoding the enzyme deoxyhypusine synthase for the genetic inactivation of the *Arabidopsis* eIF5A pathway as long as DHS is required for the first activating step by hypusination of all three eIF5A protein isoforms (Belda-Palazón et al., 2014). Since *DHS* has been shown to be essential in *Arabidopsis* (Pagnussat et al., 2005) we chose the use of conditional knock-down of *DHS* by cloning gene specific sequences into the binary vector *pOpOff2*, that allows dexamethasone-dependent induction of the RNAi pathway (Wielopolska et al., 2005). Three independent T3 homozygous transgenic lines (*H, K*, and *M*) were selected for appropriate gene silencing upon dexamethasone treatment. We checked by RTqPCR the *DHS* gene expression levels and, as it is shown in Figure 1A, an average of 85% *DHS* gene silencing was achieved after treatment of plant seedlings by induction with dexamethasone. When seeds were sown directly on medium with or without dexamethasone, the average silencing rate achieved was lower, around 50% (data not shown). As shown in Figure 1A a mild gene silencing was detected even in the absence of dexamethasone thus reflecting that the gene silencing system is slightly leaky. To further demonstrate that the genetic inactivation of *DHS* gene leads to a reduction of the DHS protein, we took advantage of a recently developed biochemical assay to monitor the hypusination status of eIF5A based on 2D electrophoresis/western blot analysis. This analysis allows the biochemical separation and quantification of the acidic and inactive non-hypusinated eIF5A proteoform from the more basic and active hypusinated proteoform (Belda-Palazón et al., 2014). Figure 1B shows around 2-fold increase of the non-hypusinated eIF5A1 proteoform in protein extracts from 10-old-day plants grown with dexamethasone compared to the mock treatment, indicating a reduction of the DHS enzyme activity in the dexamethasone treated plants. We also detected a moderate increase (1.4-fold) of a more acidic eIF5A1 proteoform in the plants treated with dexamethasone that very likely corresponds to the non-hypusinated and acetylated proteoform which is frequently observed upon *DHS* silencing in other systems (Pällmann et al., 2015). In fact non-hypusinated eIF5A has been shown to be rapidly acetylated in HeLa cells changing its subcellular localization (Lee et al., 2009; Ishfaq et al., 2012). The recent availability of a commercial antibody against hypusine allowed us to test directly the global hypusination level by means of standard PAGE/western blot analysis. Figure 1C shows that total protein samples from dexamethasone treated plants display around half of the amount of hypusine compared to untreated samples.

These data demonstrate that the transgenic plants generated for *DHS* conditional silencing (*siDHS* plants) respond to dexamethasone treatment with a partial loss of the DHS enzyme activity thus causing inactivation of eIF5A function.

### Developmental alterations of *siDHS* transgenic plants

**Figure 2:**
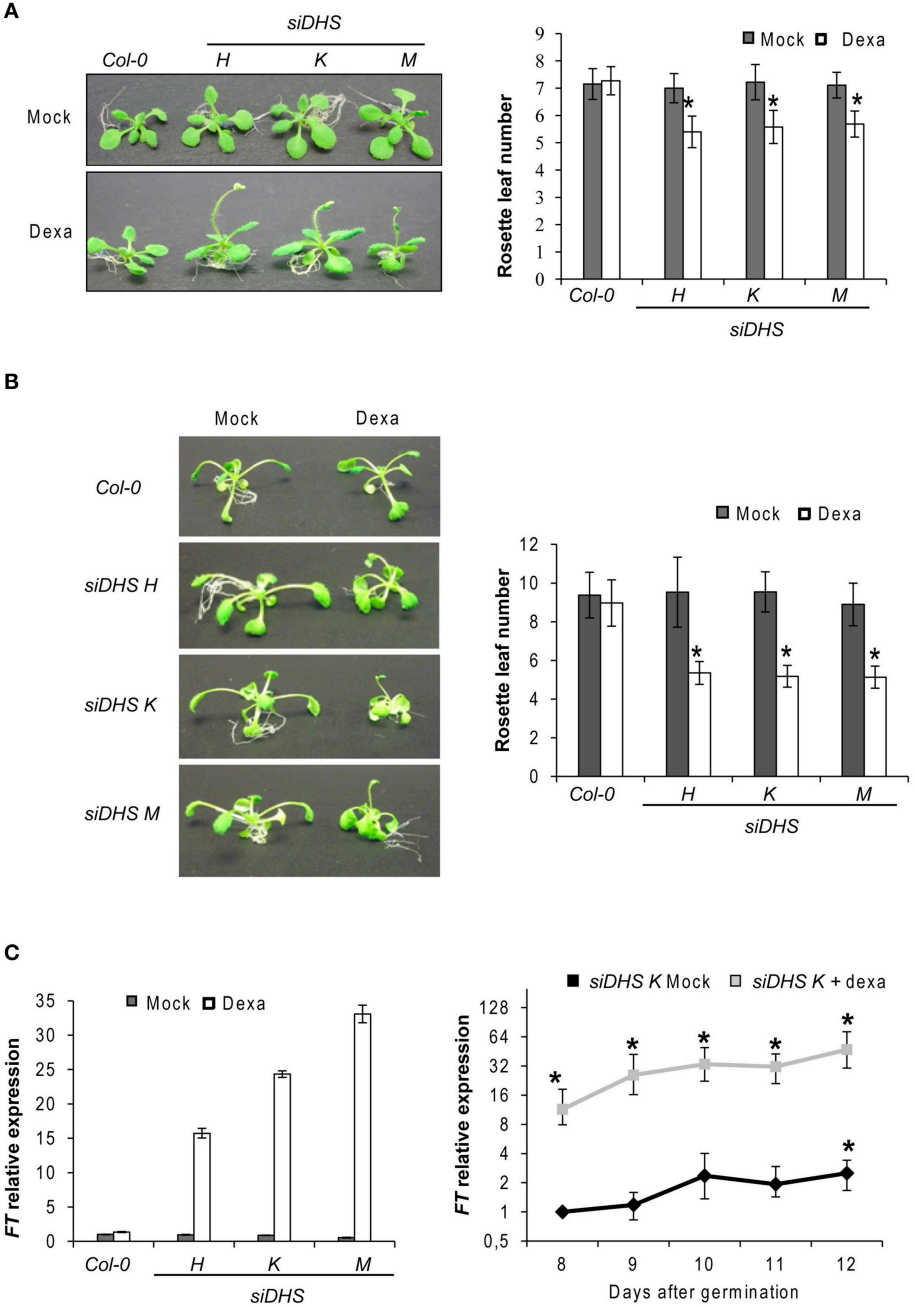
**Early flowering phenotype of *siDHS* dexamethasone-treated plants and *FT* gene induction**. **(A)** Flowering time phenotypes of representative wild type and *siDHS* plant seedlings grown *in vitro* under long-day conditions for 2 weeks with (dexa) or without (mock) dexamethasone are shown in the left side. The flowering time phenotype was quantified by counting the rosette leaf number as shown in the right side. Error bars represent the standard deviation of at least 20 plant seedlings. The data were analyzed by one-way anova test and the asterisks indicate significant differences (*p* < 0.05) with respect to wild type plants and with respect to the same mock-treated genotype. **(B)** The flowering phenotype (left) was analyzed for plants grown *in vitro* under short-day conditions plus 1% sucrose with or without dexamethasone and quantified by counting the rosette leaf number (right). Error bars represent the standard deviation of at least 20 plant seedlings. The data were analyzed by one-way anova test and the asterisks indicate significant differences (*p* < 0.05) with respect to wild type plants and with respect to the same mock-treated genotype. **(C)** *FT* gene expression relative to the mock Col-0 sample was performed by RTqPCR with 9-day-old plant seedlings treated or untreated with dexamethasone (left). Error bars represent standard deviation from three independent technical replicates. The graph on the right shows the time-course of *FT* gene expression in the transgenic line *siDHS-K* with or without dexamethasone. Error bars indicate standard deviation from three independent biological replicates and the asterisks indicate significant differences (*p* < 0.05) with respect to untreated 8-day-old wild type plants.

In the course of our initial studies of the *siDHS* plant seedlings grown *in vitro* under long-day conditions in the presence of dexamethasone, we observed a consistent early flowering phenotype as it is shown in Figure 2A (left). The phenotype was confirmed when counting the rosette leaf number of *siDHS* plants treated with dexamethasone as they bolted with 4–6 rosette leaves after 11–12 days compared to the 6–8 rosette leaves after 16–18 days for the wild type and untreated transgenic plants (Figure 2A, right). *siDHS* plants treated with dexamethasone also displayed an early flowering phenotype when grown *in vitro* under short-day conditions as it is shown in Figure 2B. To accelerate plant growth under short-day conditions thus facilitating the counting of rosette leaves as a measure of flowering time, we grew the plants *in vitro* in the presence of 1% sucrose. As it is shown in Figure 2B the *siDHS* plants treated with dexamethasone bolted after 16–17 days with 4–6 rosette leaves compared to 30–32 days and 8–10 leaves for the untreated transgenic and wild type plants. In addition to the early flowering phenotype *siDHS* plants treated with dexamethasone displayed growth alterations under short-day conditions in the presence of 1% sucrose as they had shorter and curved petioles whereas wild type and the transgenic plants without dexamethasone had elongated and straight petioles. To study the molecular basis of the early flowering phenotype we analyzed by RTqPCR the expression of some key genes involved in the transition process, such as *FT, FLC*, and *CO*. Plant samples were taken from 9-day-old plant seedlings 4 h after subjective dawn under long-day conditions to avoid circadian-dependent variations on gene expression. Whereas *FLC* and *CO* did not show any significant alteration (data not shown), *FT* gene expression showed dramatic alterations of around 20-fold increases on average for all *siDHS* plants treated with dexamethasone (Figure 2C, left panel). The *FT* gene expression time-course was studied for *siDHS* plants grown *in vitro* under long-day conditions with and without dexamethasone during the critical period of phase transition (days 8–12). The dexamethasone treatment caused a sustained increase on *FT* gene expression from around 10-fold at day 8, up to 50-fold at day 12 when compared to untreated *siDHS* plants as it is shown in Figure 2C (right panel). Lack of dexamethasone treatment showed the usual *FT* gene induction of about 3-fold in the same developmental period. To further check the dependence of the FT activity on the early flowering phenotype of *siDHS* plants treated with dexamethasone, we introduced the *siDHS* silencing constructs into the *ft-10* mutant genotype. The T3 homozygous *siDHS* plants on *ft-10* background also responded to *DHS* silencing upon dexamethasone treatment but did not show any early flowering phenotype and they behaved as late flowering plants. Hence the *ft* mutation shows epistasis over *DHS* silencing (Figure S1). Altogether the data indicate that knock-down of *DHS* leads to accelerated flowering by enhancing *FT* mRNA levels.

**Figure 3:**
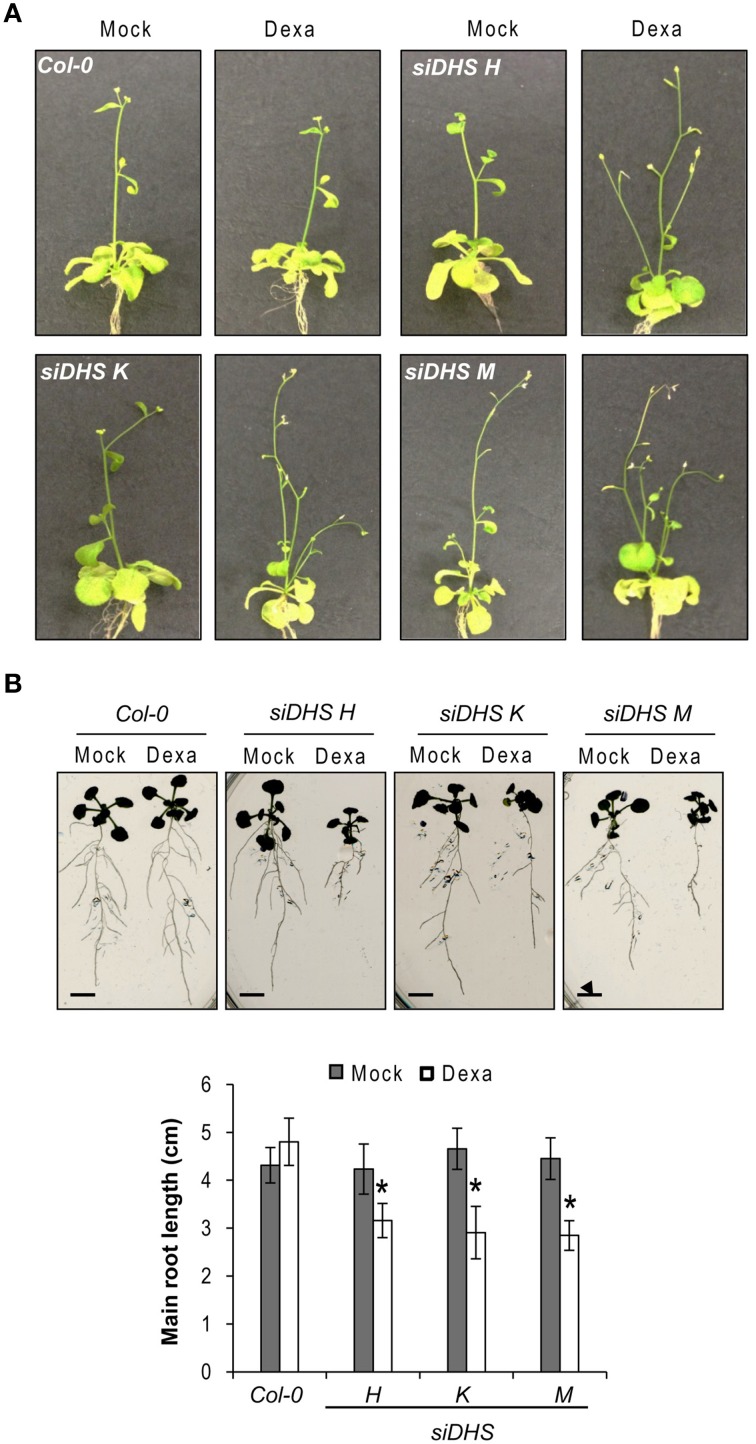
**Developmental alterations in shoot and root growth upon *DHS* silencing. (A)** Representative images of 25-day-old wild type and *siDHS* transgenic plants grown *in vitro* under long-day conditions with (dexa) or without (mock) dexamethasone showing a bushy phenotype when *DHS* is silenced. **(B)** Root growth was analyzed on 14-day-old wild type and *siDHS* plants treated or not with dexamethasone. A representative image shows root growth defects upon dexamethasone treatment for *siDHS* plants. The length of the main root of at least 5 individual plants of each transgenic line and treatment was quantified with ImageJ and the results are shown below. Asterisks indicate significant differences (*p* < 0.05) with respect to wild type plants and with respect to the same mock-treated genotype. Scale bars represent 0.5 cm.

**Figure 4:**
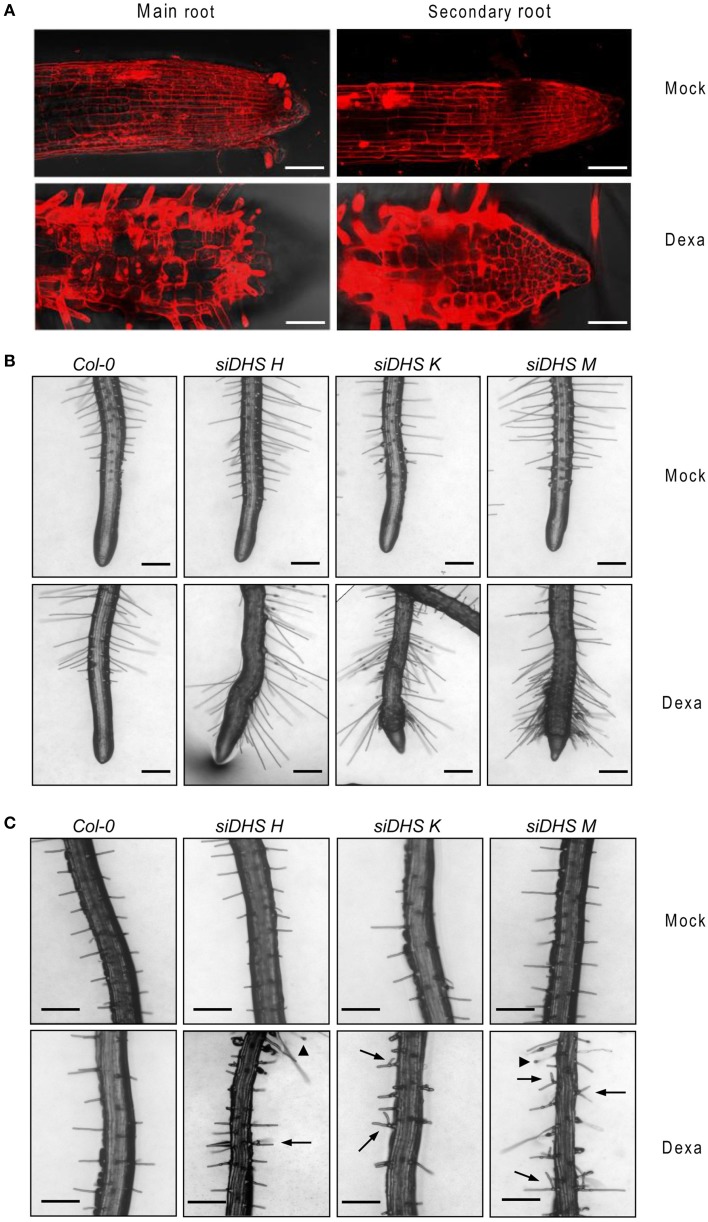
**Alterations in root cell expansion and root hair phenotypes caused by *DHS* silencing. (A)** Confocal microscope images after propidium iodide staining of primary and secondary roots of *siDHS-M* plants grown *in vitro* for 18 days under long-day conditions with (dexa) or without (mock) dexamethasone. Scale bars represent 50 μm. **(B)** Root hair pictures of 14-day-old wild type and *siDHS* transgenic plants grown *in vitro* under long-day conditions with (dexa) or without (mock) dexamethasone were taken close to the tip of secondary roots. **(C)** Root hair pictures were taken from the intermediate region of primary roots of the same plants as in **(B)**. Arrowheads and arrows mark abnormal tip swelling and branched root hairs, respectively, in silenced *DHS* plants. Scale bars represent 0.2 mm.

The conditional *DHS* knock-down also caused changes on plant architecture in addition to the alterations in petiole growth shown in Figure 2B. Figure 3A shows that dexamethasone treatment of *siDHS* plants grown under long-day conditions produced an increase in shoot branching, a “bushy” phenotype that resembles the loss-of-function of *BUD2* encoding an S-adenosylmethionine decarboxylase involved in spermidine biosynthesis (Ge et al., 2006). In addition to shoot architecture, roots of *siDHS* plants treated with dexamethasone displayed growth inhibition detectable after 12 days (Figure 3B) when compared to wild type and untreated plants. The main root of *siDHS* plants in the presence of dexamethasone grew up to 3 cm on average after 14 days, whereas wild type and untreated plants could grow up to 4.5 cm on average in the same period of time. To further investigate the phenotype of root growth inhibition we microphotographed propidium iodide stained roots of *siDHS-M* plants grown with or without dexamethasone (Figure 4A). Both main and secondary roots of dexamethasone-treated plants displayed aberrant cell morphology behavior in the tip, with apparent cell elongation defects potentially explaining the alterations in root growth. Consequently, the differentiation zone where root hairs initiate appeared closer to the root tip in dexamethasone-treated seedlings, as shown in Figure 4B. Furthermore, some root hairs appeared abnormal in dexamethasone-treated *siDHS* roots, displaying tip swelling and aberrant branching (Figure 4C). Altogether the data indicate that both shoot and root architecture are altered upon *DHS* gene silencing.

### Hypersensitivity of *siDHS* transgenic plants to osmotic stressors and ABA

**Figure 5:**
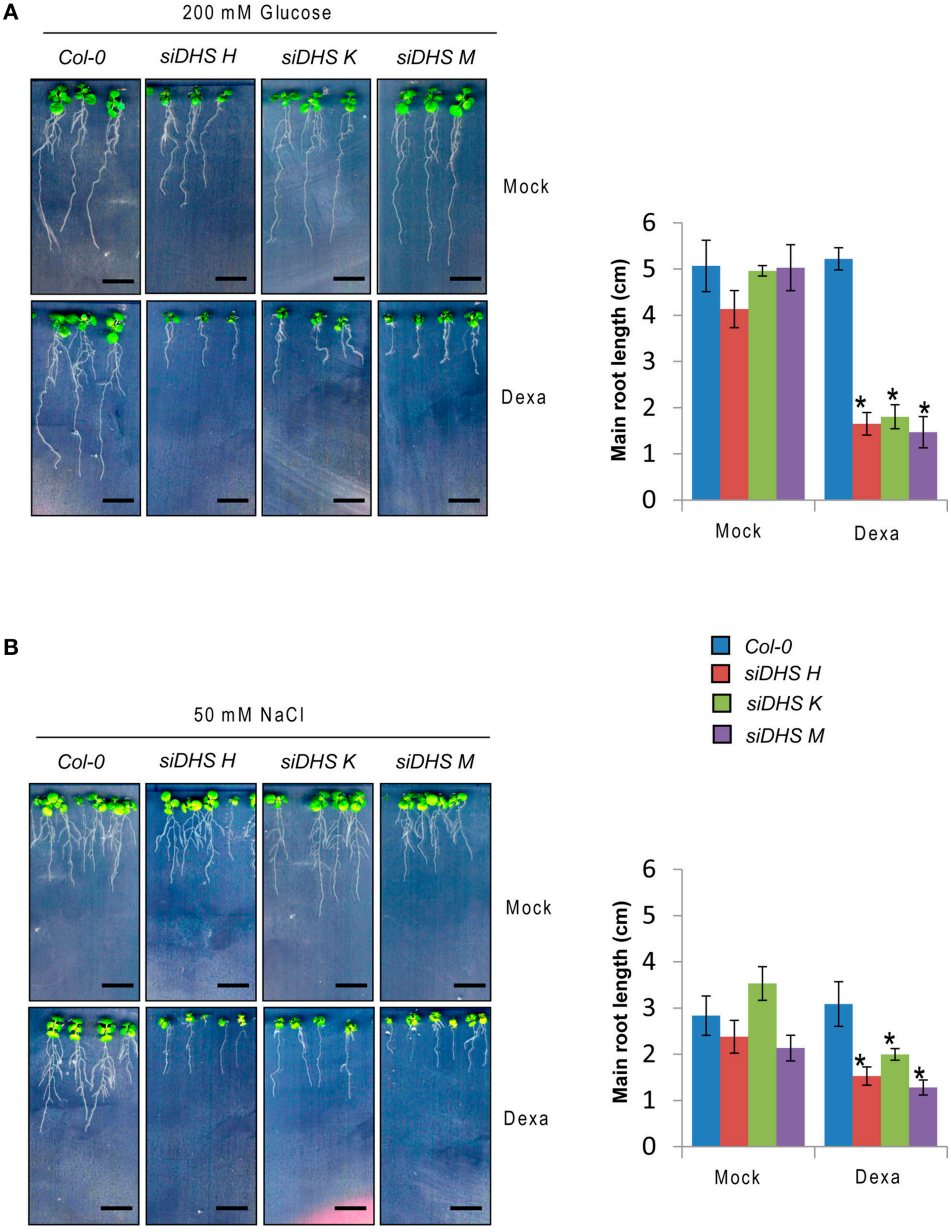
***DHS* silencing leads to glucose and NaCl hypersensitivity. (A)** 4-day-old plants grown *in vitro* under long-day conditions with or without dexamethasone were transferred to medium with 200 mM D-glucose for 10 additional days when pictures were taken (left) and the growth of the main root was measured (right). **(B)** Plants were sown directly on medium with or without 50 mM NaCl in the presence or absence of dexamethasone, and the root growth was recorded (left) and measured (right) at the age of 14-day-old plants. In all the cases the length of the main root of at least 5 individual plants of each transgenic line and treatment was quantified with ImageJ. Asterisks indicate significant differences (*p* < 0.05) with respect to mock-treated plants of the same genotype. Scale bars represent 0.5 cm.

**Figure 6:**
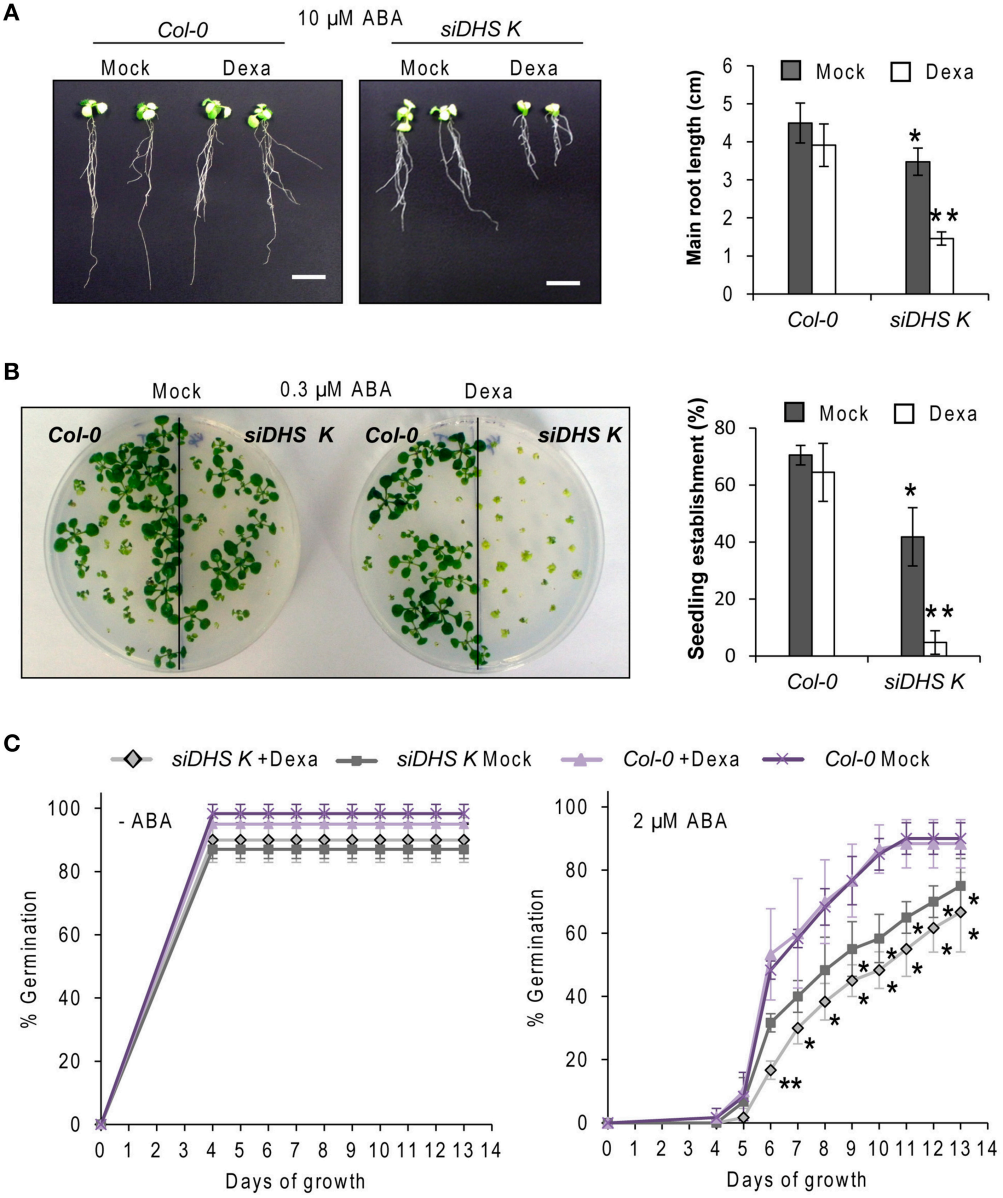
***DHS* silencing causes hypersensitivity to ABA. (A)** 4-day-old wild type and *siDHS-K* plants grown *in vitro* under long-day conditions with or without dexamethasone were transferred to medium with 10 μM ABA for 10 additional days when pictures were taken (left) and the growth of the main root of at least 5 individual plants of each transgenic line and treatment was measured (right). **(B)** Seedling establishment was estimated by counting wild type and *siDHS-K* plant seedlings with open healthy leaves after sowing in the absence or in the presence of 0.3 μM ABA with or without dexamethasone. Left panel shows a representative picture and the statistical quantification with at least 6 individual plants of each genotype and treatment is shown on the right. **(C)** Germination percentage was determined by counting the number of seedlings able to culminate radicle protrusion after sowing in the absence or in the presence of 2 μM ABA with or without dexamethasone. The statistical analysis performed in all the cases was one-way anova test. Asterisks indicate significant differences (*p* < 0.05) with respect to wild type plants (one asterisk) or with respect to wild type plants and mock-treated plants of the same genotype (two asterisks).

As the eIF5A pathway has been involved in the *Arabidopsis* response to osmotic and nutrient stresses (Wang et al., 2003; Ma et al., 2010) we checked the behavior of *siDHS* transgenic plants under sub-lethal concentrations of osmotic stressors glucose and NaCl, and the stress hormone ABA. We chose the root growth inhibition of 14-day-old seedlings as a parameter to quantify growth responses since we had previously calibrated the component of root growth inhibition due to the conditional inactivation of the eIF5A pathway on plants of the same age as it is shown in Figure 3B. Under sub-toxic concentrations of Glucose (200 mM) neither wild type nor dexamethasone-free transgenic plants displayed any reduction in root growth but even a subtle growth enhancement up to 5 cm length on average. However, the presence of dexamethasone on *siDHS* plants treated with glucose provoked a stronger root growth inhibition than the simple addition of dexamethasone, with a maximum root length of 1.5 cm on average (Figure 5A). Similarly sub-lethal 50 mM NaCl concentration caused increased root growth inhibition compared to normal medium for *siDHS* plants with dexamethasone with a maximum root length up to 1.5 cm on average. In this last case the differences were more subtle as both wild type and not silenced *siDHS* plants displayed partial growth inhibition under salt treatment with a maximum root length of 3 cm on average (Figure 5B). A similar growth inhibition response was observed with sub-toxic ABA concentrations. In this case we used only the *siDHS* transgenic line K as different studies of ABA responses were performed, namely root growth inhibition, seedling establishment and germination rate. The presence of 10 μM ABA in the medium for a short period did not inhibit root growth of wild type plants, whose main roots were capable of growing up to 4.5 cm length on average. However, the *siDHS*-K plants not-treated with dexamethasone already displayed a partial root growth inhibition that was greatly enhanced upon dexamethasone treatment with a maximum root length of 1.5 cm (Figure 6A). The sensitivity of transgenic *siDHS* plants to ABA in the absence of the silencing drug dexamethasone probably reflects that the genetic inactivation system is slightly leaky and that minor inactivation of the eIF5A pathway is very sensitive to alterations in ABA homeostasis. A similar result of enhanced sensitivity to ABA upon *DHS* silencing was obtained when the percentage of seedling establishment was recorded for *siDHS*-K plants treated or not with dexamethasone in the presence of 0.3 μM ABA, as shown in Figure 6B. Finally, the percentage of germination in the presence or absence of 2 μM ABA was also tested showing both a basal hypersensitive response to ABA of *siDHS* plants in the absence of dexamethasone and an enhanced ABA response upon dexamethasone treatment mostly significant at day 6 (Figure 6C).

To summarize, all the data presented indicate that *DHS* silencing leads to enhanced sensitivity of *Arabidopsis* plants to challenging growth conditions by the presence of glucose or salt and by the exogenous application of the stress hormone ABA.

### Identification and analysis of protein interaction networks and biological processes for polyproline-rich proteins

**Figure 7:**
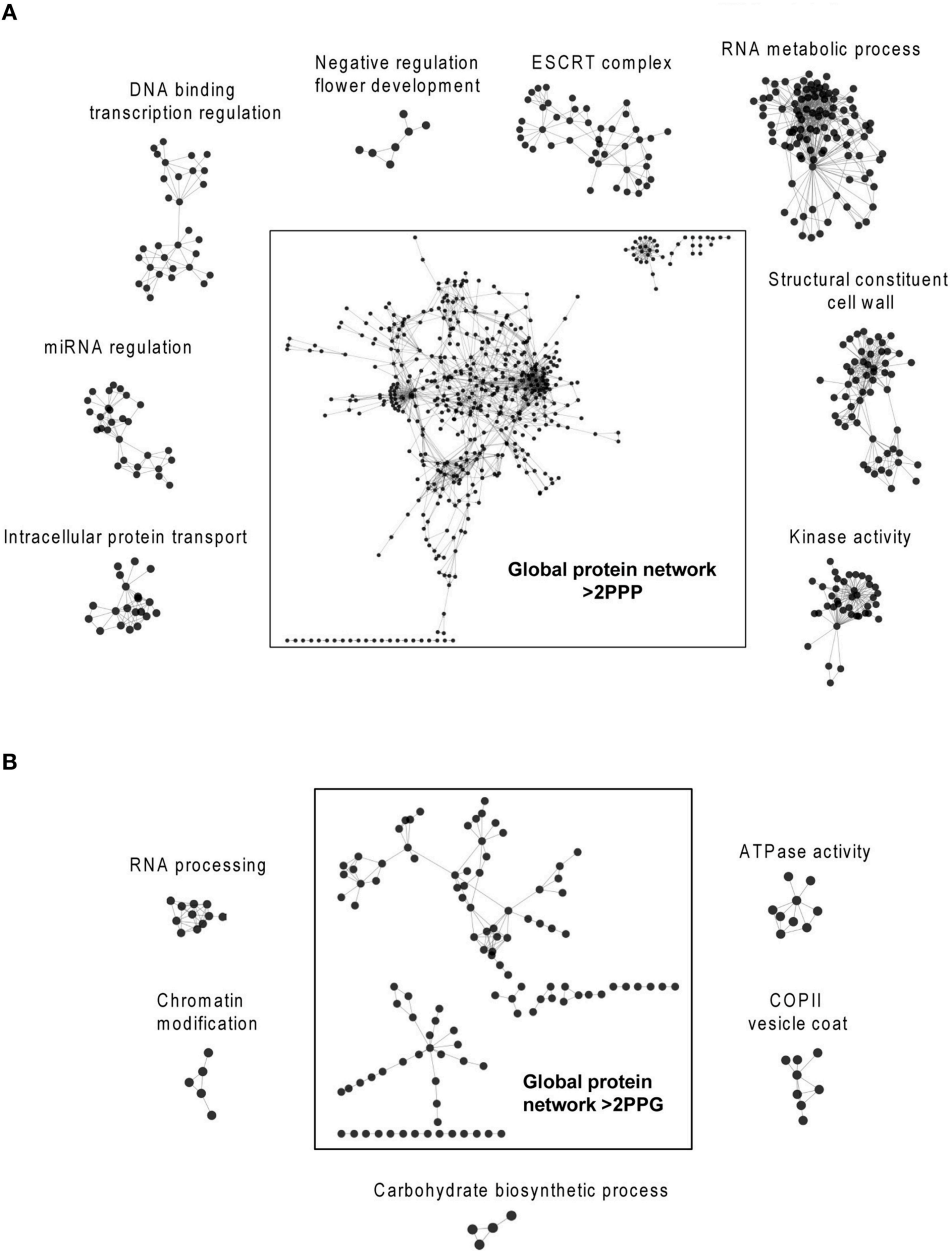
**Bioinformatic search of polyproline-rich protein network clusters**. The *Arabidopsis* lists of proteins with >2 PPP units **(A)** or >2 PPG units **(B)** were analyzed with the String web-based tool and visualized with the program Cytoscape. The Cytoscape plugins MCODE and BiNGO were used to identify protein clusters and to determine their gene ontology, respectively. The highest scoring clusters are represented surrounding the global networks. Additional information on the global gene ontology data for both protein networks are shown in Tables S1, S2.

The recent discovery that eIF5A and its functional homolog EFP in eubacteria are both required for efficient translation of mRNAs encoding proline rich-repeat proteins (Doerfel et al., 2013; Gutierrez et al., 2013; Ude et al., 2013) has opened the path to identify potential eIF5A mRNA clients through bioinformatic approaches (Mandal et al., 2014; Pällmann et al., 2015). The rationale behind these studies is that if polyproline-rich proteins considered as eIF5A targets arrange as clusters of protein-protein interaction networks, one may envisage the biological functions and evolutive specialization of the axis spermidine/eIF5A in the organism of interest by studying the topology and ontology of the protein network. Ideally the knowledge of the network organization will fit the phenotypic data generated by the genetic inactivation of the pathway that should disrupt protein complexes whose stoichiometry depends on a functional eIF5A, and thus help identify potential eIF5A mRNA clients. With these ideas in mind we interrogated the *Arabidopsis* genome encoding proteins with >2 PPP (857 proteins) or >2 PPG (172 proteins) repeats per protein according to (Mandal et al., 2014) for their organization as a network of interacting proteins with the use of the web tool STRING and the Cytoscape software for visualization and cluster identification with MCODE and BinGO plugins. We decided to use >2 PPP or >2 PPG instead of >1 PPP or >1 PPG, as it was done in mouse, since the protein list in *Arabidopsis* with >1 PPP (3503 proteins) almost doubles the number of mouse and exceeded the gateway web working process of the STRING algorithm. The bioinformatic analysis with the STRING web-based tool yielded one protein interaction network for proteins containing >2 PPG and another for the protein list with >2 PPP. The topological parameters of the networks identified are shown in Table 2, and their values are similar to the murine and yeast protein networks recently published (Pällmann et al., 2015). The identified protein interaction networks were interrogated with the MCODE plugin for the presence of densely connected sub-networks (clusters) that may represent arrangement of protein complexes. A total of 21 and 6 clusters were identified for PPP-rich and PPG-rich protein networks, respectively. The clusters identified with MCODE, as well as the global protein networks, were analyzed with the BiNGO tool to predict their biological functionality and the results of the highest scoring of the gene ontology (GO) terms are shown in Figure 7 for the clusters and on Tables S1, S2 for the global networks. Although not every identified cluster could be assigned to a functional group by GO enrichment, those that could be identified, together with the GO terms of the global networks, revealed important information when compared to the murine and yeast data. On the one hand some GO terms are highly conserved among all three eukaryotes studied as it is the case of the processes related to mRNA metabolism, regulation of transcription and organization of the cytoskeleton. On the other hand several GO terms are shared only between mouse and *Arabidopsis* such as cell differentiation and chromatin modification. Finally a number of GO terms show up specifically for *Arabidopsis* such as cell wall constituents, kinase activities, intracellular protein transport, regulation of flowering, regulation of circadian rhythm, miRNA regulation, ATPase activity, and carbohydrate biosynthesis. Overall these bioinformatic studies suggest that there is a well-oriented functional specialization for polyproline-rich proteins when comparing the GO term enrichment distribution of protein interaction networks among yeast, mouse and plants, thus uncovering a highly specialized function for the axis spermidine/eIF5A throughout the eukaryotic evolution.

**Table 2:** **Protein network topology parameters**.

**Network parameters**	**>2PPP unit**	**>2PPG unit**
Number of proteins for network construction with STRING	857	172
Number of nodes	486	106
Clustering coefficient	0.281	0.179
Average number of neighbors	6.025	2.377
Network heterogeneity	1.182	0.862

## Discussion

The role of spermidine as a chemical donor of the aminobutyl moiety used for the enzymatic hypusination of the essential translation factor eIF5A highlights its importance. The activity of eIF5A within the ribosome as a non-canonical translation elongation factor in yeast (Saini et al., 2009) and the essential function attributed to eIF5A for the translation of mRNAs that encode proline rich-repeat proteins (Doerfel et al., 2013; Gutierrez et al., 2013; Ude et al., 2013) has led to the very exciting hypothesis that the axis spermidine/eIF5A may have evolved together with the functional specialization of polyproline-rich proteins. To further elaborate on this hypothesis, it is necessary to understand the organization and function of polyproline-rich proteins within the eukaryotic proteomes.

In this work we have shown that alterations in the biosynthesis of hypusine promoted by the DHS inactivation in *Arabidopsis*, result in a wide variety of phenotypes affecting many biological processes related with development (control of flowering time, the aerial and root architecture and root hair phenotypes) and with the adaptation to challenging growth conditions (presence of salt, glucose and ABA in the growth medium), raising the question on how eIF5A might be involved in those plant responses. To find molecular explanations for the phenotypic data described we have tested with bioinformatic approaches the hypothesis that the phenotypes caused by *DHS* silencing might be correlated with functional organization of proline rich-repeat proteins as potential targets of the axis spermidine/eIF5A. It is remarkable that in addition to common putative protein complexes to those identified in yeast and mice, *Arabidopsis* also contains specific polyproline-rich protein complexes that may have evolved for adaptation to particular plant functionalities. These data support the idea of an evolutive co-specialization of the spermidine/eIF5A pathway and the organization of proline rich-repeat proteins. Interestingly some of the biological functions unveiled by the identification of GO terms of PPP-rich and PPG-rich protein interaction networks in *Arabidopsis* may explain some of the phenotypes identified by *DHS* inactivation. This is the case of the early flowering phenotype since a specific protein cluster was found in the bioinformatic search that includes potential eIF5A targets whose defects during translation may lead to an early flowering phenotype. Among the protein clusters with common ontology to other eukaryotic organisms, the organization of the actin cytoskeleton may explain some of the root growth and root hair phenotypes revealed in this work. First it was previously reported that major rearrangements of actin microfilaments (F-actin) occur in the transition zone between the meristem and the elongation/differentiation zone in the root (Baluska et al., 1997) and the blockade of actin polymerization with pharmacological inhibitors led to defects in root cell elongation (Baluška et al., 2001). Our observations of root growth defects caused by *DHS* silencing may be related to difficulties in F-actin polymerization as suggested by the alterations in root cell elongation close to the tip. In addition, this alteration in root cell elongation may explain the abnormal accumulation of root hairs close to the tip of *DHS* silenced plants. On the other hand we have also disclosed root hair phenotypes in the proximal differentiated zone that may be explained by F-actin defects, since the alterations in root hair development with the presence of swelling tips and branched root hairs is characteristic of mutants in the polyproline-rich protein formin involved in actin polymerization (Yi et al., 2005). It is remarkable that formin protein responsible of unrelated phenotypes such as plant root hair development and polarized yeast cell growth (Li et al., 2014) may share common underlying molecular mechanisms for translation as the conserved axis spermidine/eIF5A. Other molecular explanations for the developmental alterations or for the hypersensitivity to ABA and osmotic stressors due to *DHS* inactivation may hide on the vast list of proline rich-repeat proteins whose unequivocal assignment as *bona fide* eIF5A clients requires additional experimental validation.

One main reason of the requirement for independent validation of translational alterations has emerged after the recent reports on EFP activity. The initial excitement upon the discovery of the role of EFP and eIF5A on the translation of polyproline-rich proteins has given way to a cautious optimism when the experimental data based on ribosome profiling data with *efp* mutants has shown that only a fraction of genes with pausing motifs had reduced ribosome density (Elgamal et al., 2014; Woolstenhulme et al., 2015). The authors identified several *cis* features that can modulate the strength of the ribosomal pause depending on the context, the position and the translational efficiency of the given gene. Under the light of these recent reports it becomes evident that there is a need to empirically assess the position and strength of the translational pauses on mRNAs upon inactivation of the eIF5A pathway. The powerful technique of ribosome footprint profiling (Ingolia et al., 2009) emerges as a goldmine to identify genuine eIF5A mRNA clients with unprecedented precision, as its use in plants has already been implemented (Juntawong et al., 2014). Its use after genetic or pharmacological alterations of the eIF5A pathway in *Arabidopsis* is in the pipeline, and it will provide unequivocal molecular evidences of the ribosomal roles for the axis spermidine/eIF5A during plant growth and development with the identification of authenticated eIF5A mRNA targets during translation.

Finally we should not forget that some of the phenotypic alterations caused by *DHS* inactivation may be unrelated to eIF5A ribosomal function. For instance, shoot and root alterations have been correlated with alterations in auxin and cytokinin homeostasis for mutants defective in spermidine biosynthesis (Cui et al., 2010) and are similar to some of the phenotypes reported in this work. Related to this, a recent study has assigned an atypical role to the *Arabidopsis* eIF5A2 isoform as part of the cytokinin receptor machinery that controls cytokinin signaling activity involved in development of root vasculature (Ren et al., 2013). Additional molecular actions out of the ribosome but related to mRNA life have been also attributed to eIF5A in other eukaryotic systems, such as its involvement in mRNA nucleocytoplasmic transport (Zuk and Jacobson, 1998). Therefore, it seems that the ancient axis spermidine/eIF5A has assumed central biological functions as well as specific functionalities in the evolutive adaptation of the eukaryotic cell that still remain to be carefully detailed in most of the organisms and in particular in plants.

## Author contributions

BB and AF conceived and designed the experiments. BB, CA, and EM performed the experiments. BB, CA, EM, JC, and AF analyzed the data. JC and AF wrote the paper.

### Conflict of interest statement

The authors declare that the research was conducted in the absence of any commercial or financial relationships that could be construed as a potential conflict of interest.
