# Supplementary figures and images for: Relevance of the Axis Spermidine/eIF5A for Plant Growth and Development
Source: Front Plant Sci. 2016 Mar 2;7:245. doi: 10.3389/fpls.2016.00245 (PMC4773603; doi:10.3389/fpls.2016.00245)

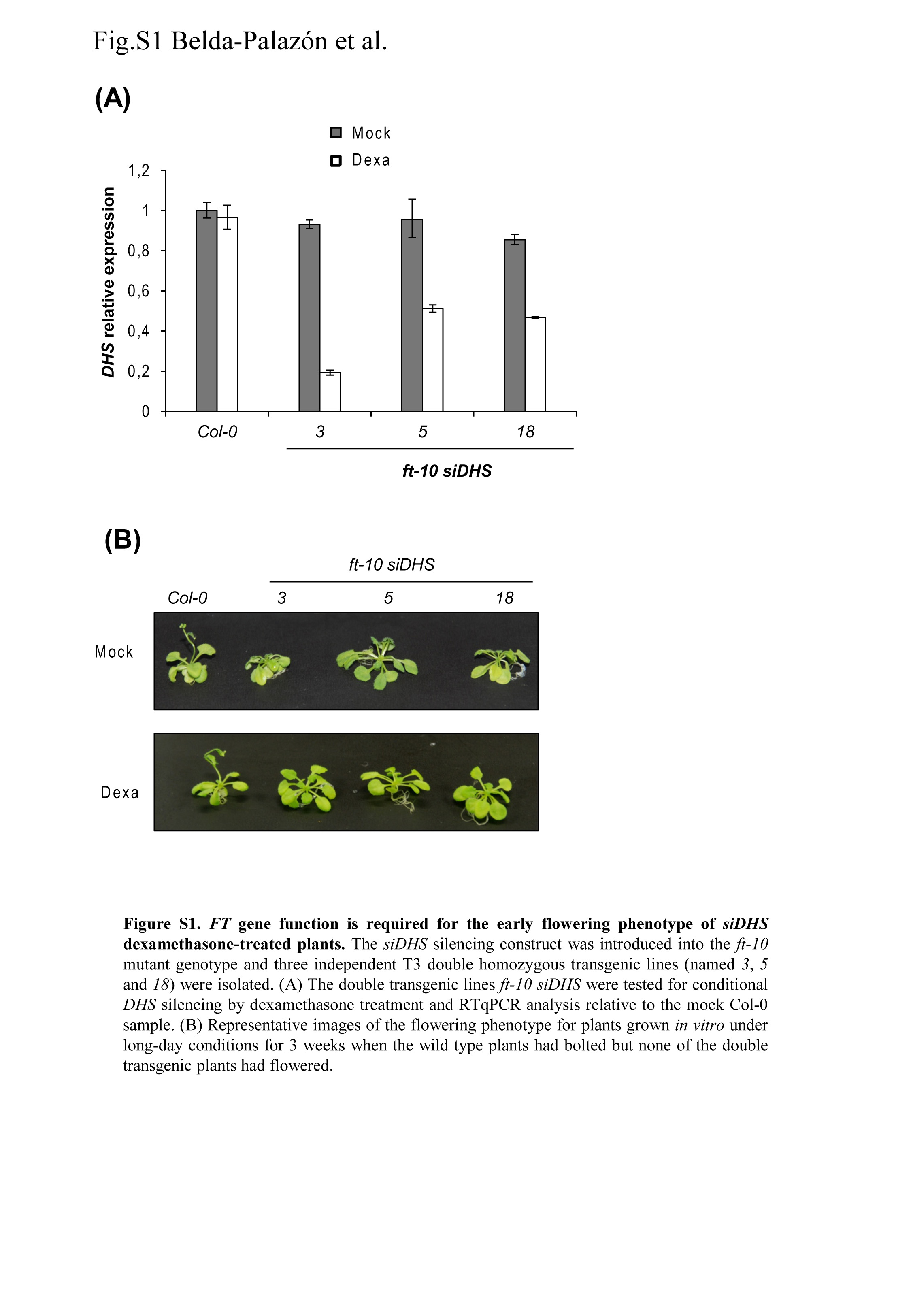

Supplement: Supplementary file 3 [file Image7.JPEG]
